# Supplementary figures and images for: Inference of recombination maps from a single pair of genomes and its application to ancient samples
Source: PLoS Genet. 2019 Nov 14;15(11):e1008449. doi: 10.1371/journal.pgen.1008449 (PMC6879166; doi:10.1371/journal.pgen.1008449)

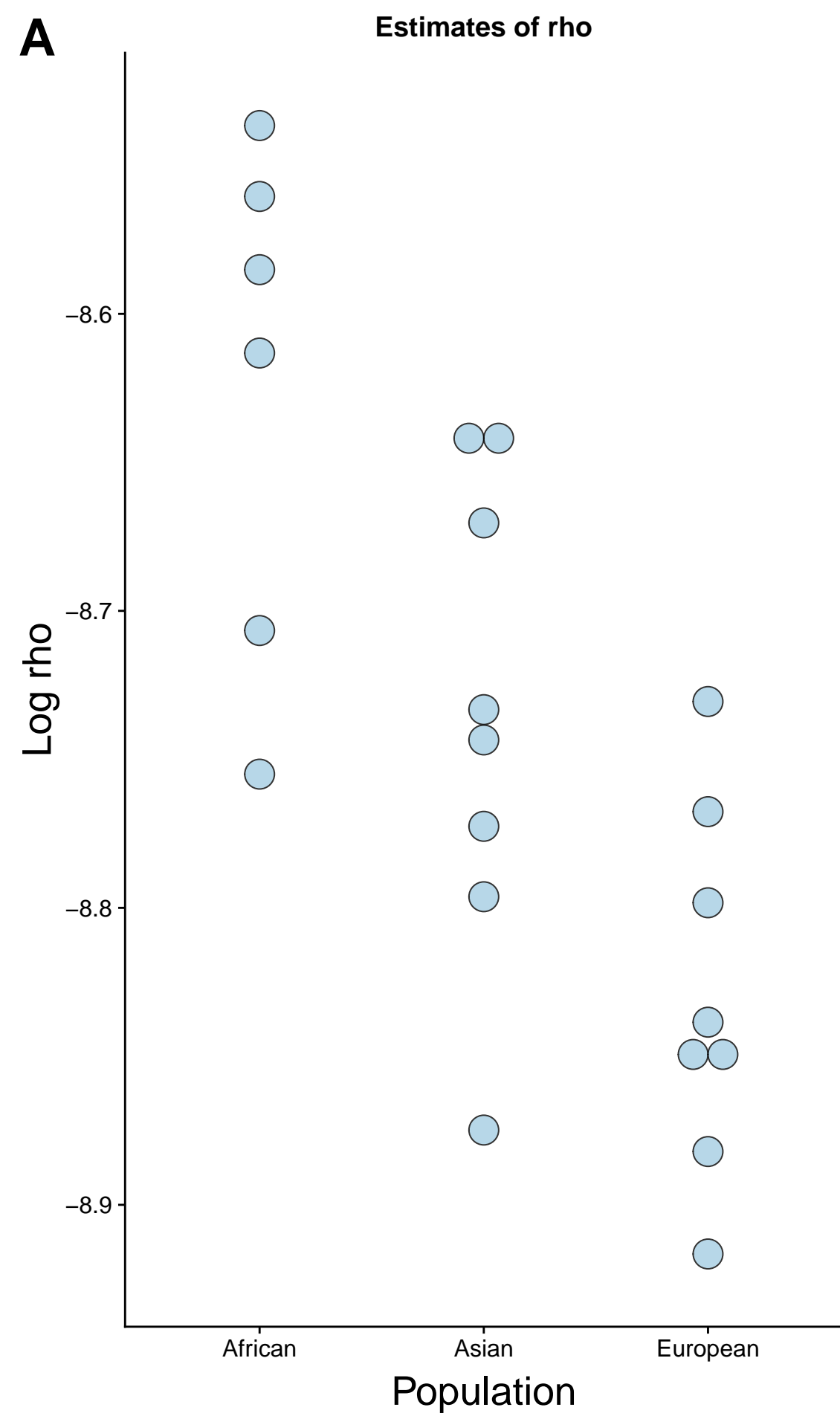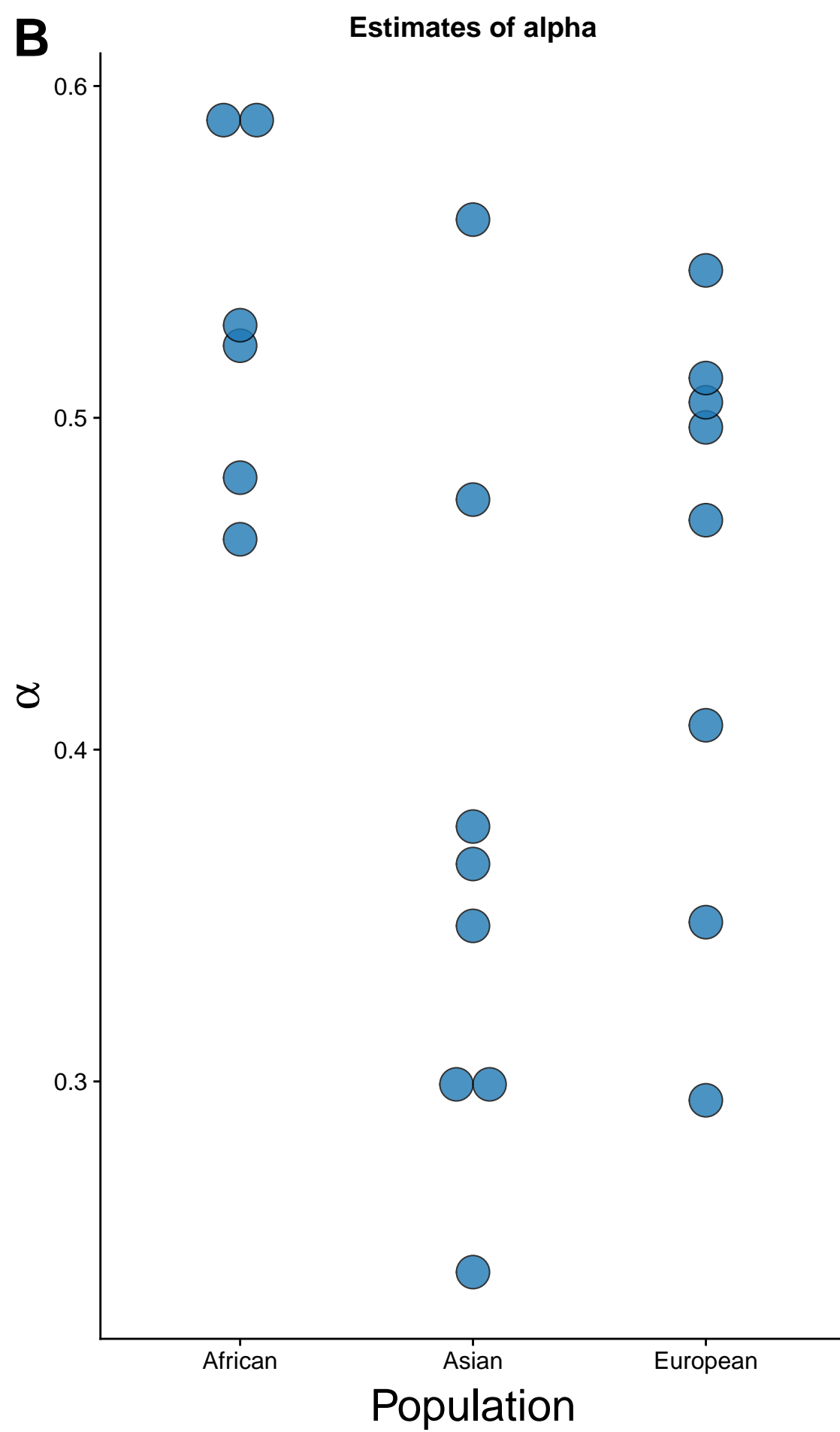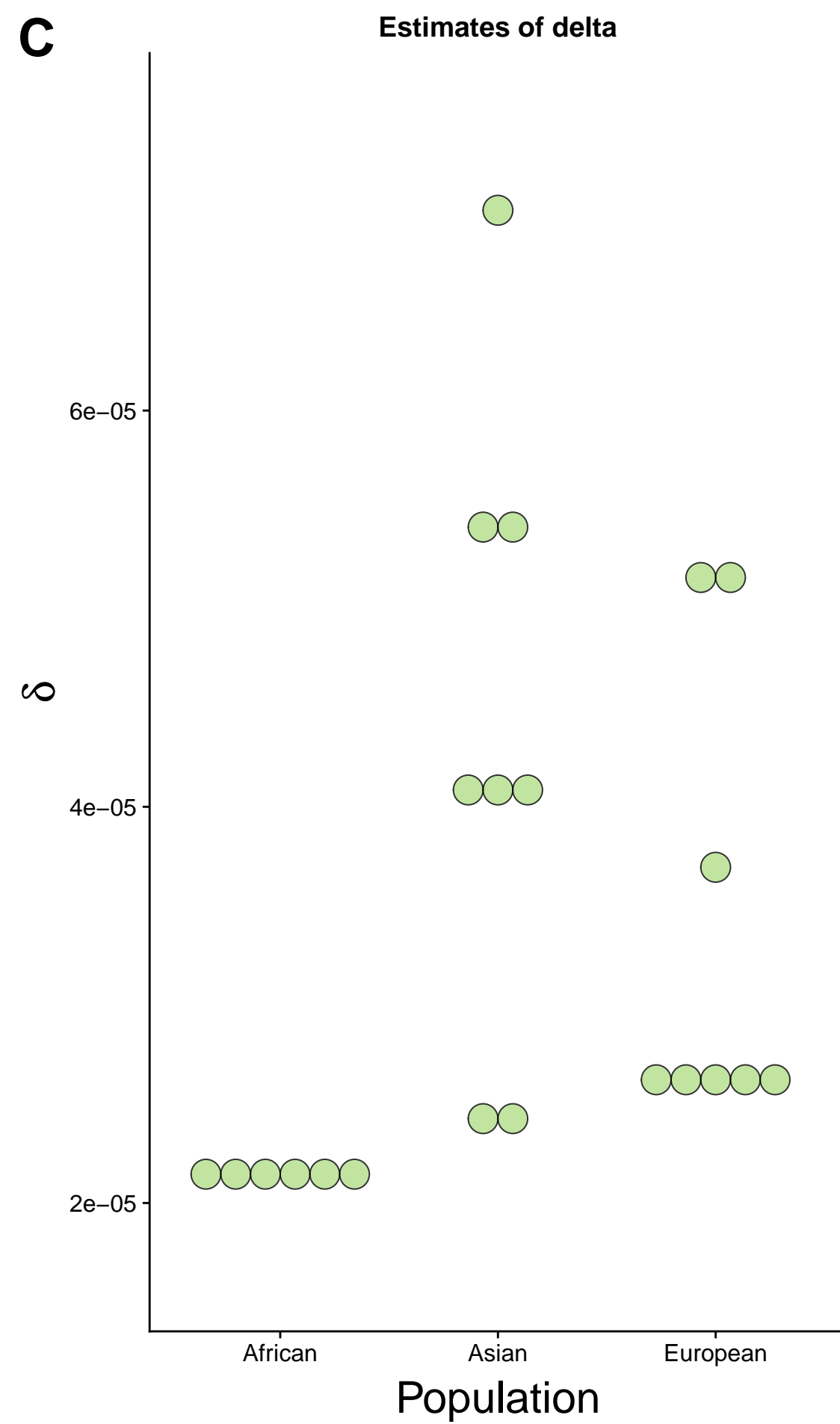

Supplement: S1 Fig — A: the genome-wide recombination rate (ρ); B: the shape of the Gamma distribution (α); C: the average frequency of change in the recombination rate along the genome (δ). (PDF) [file pgen.1008449.s007.pdf]

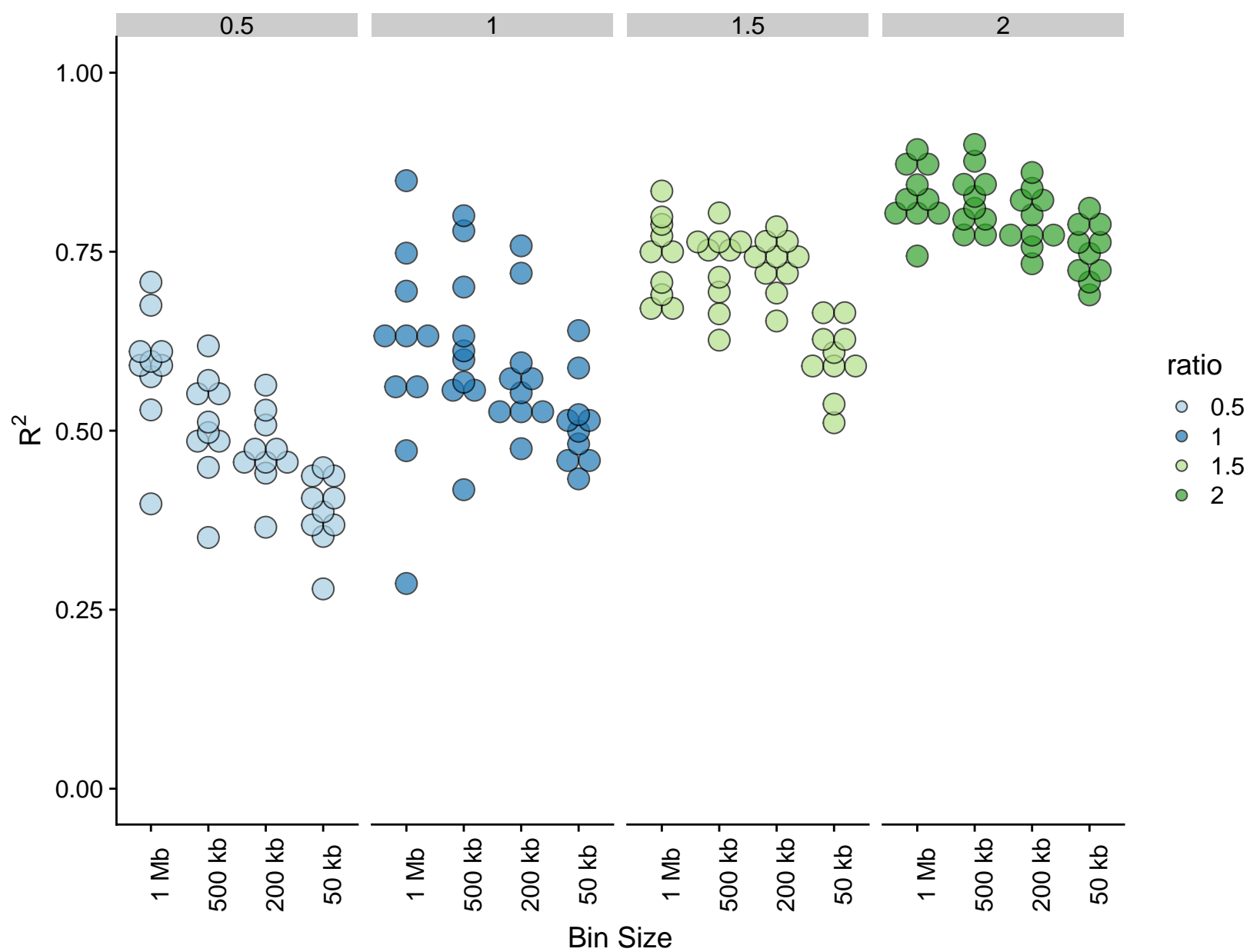

Supplement: S2 Fig — (PDF) [file pgen.1008449.s008.pdf]

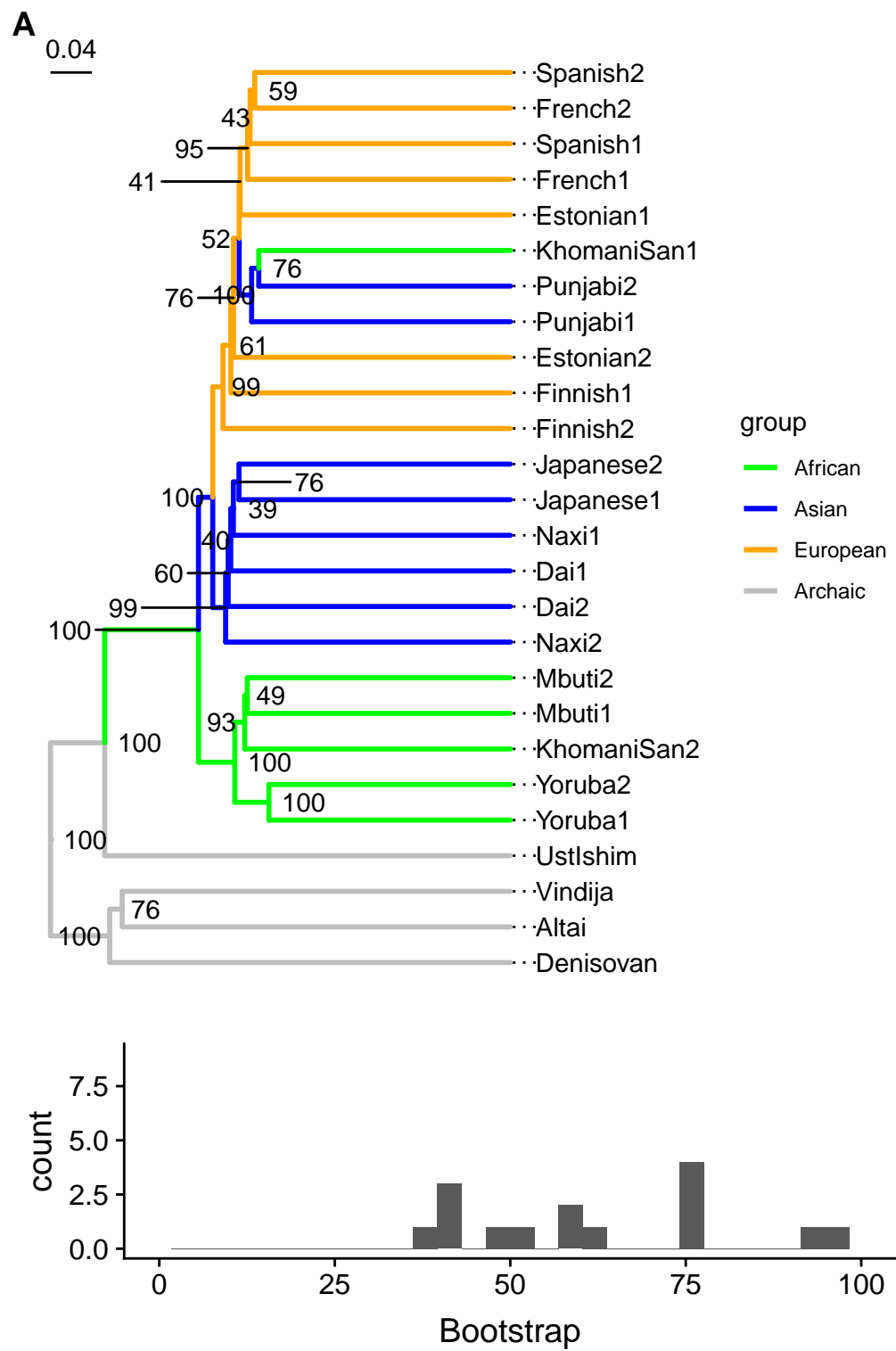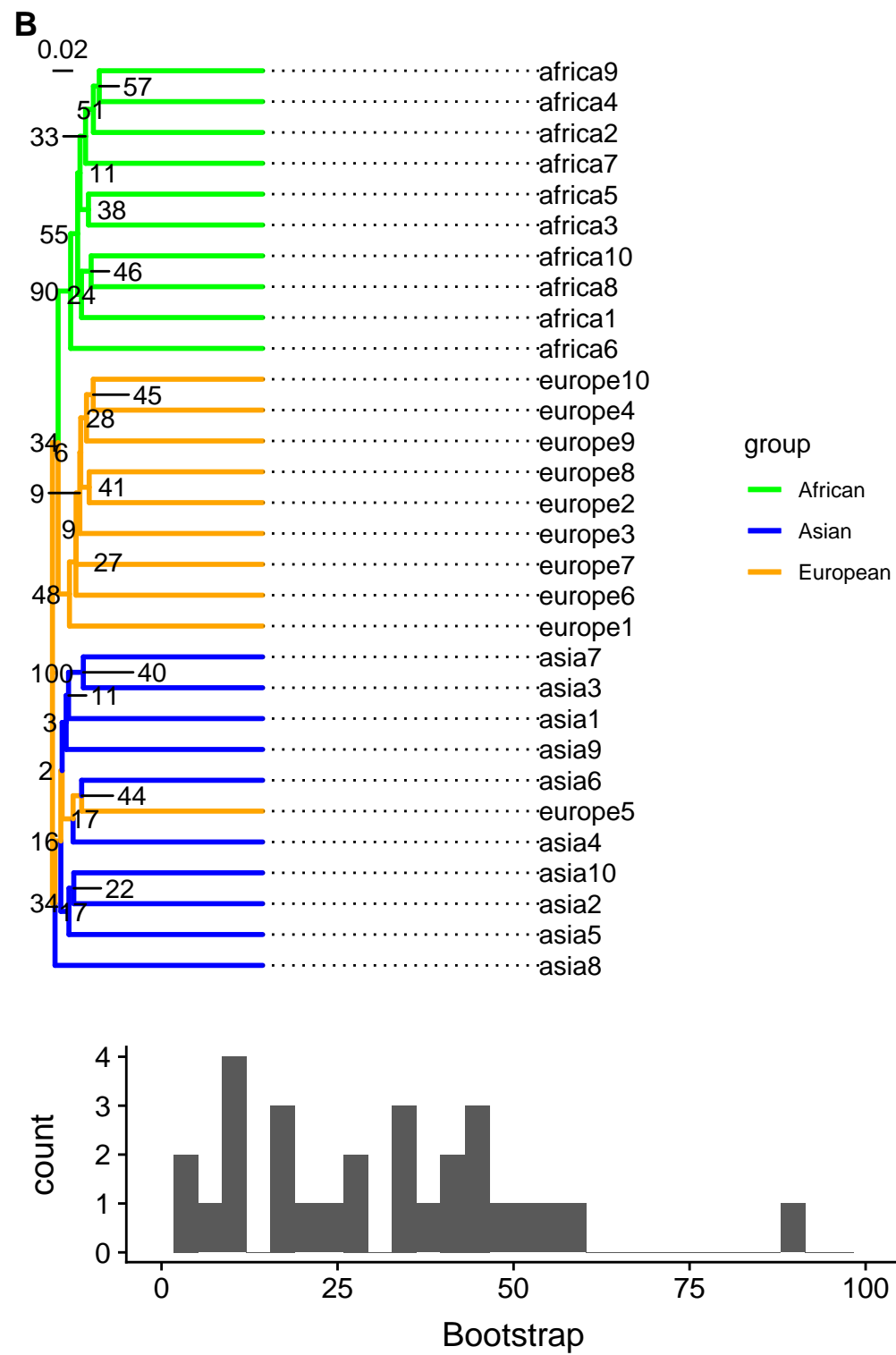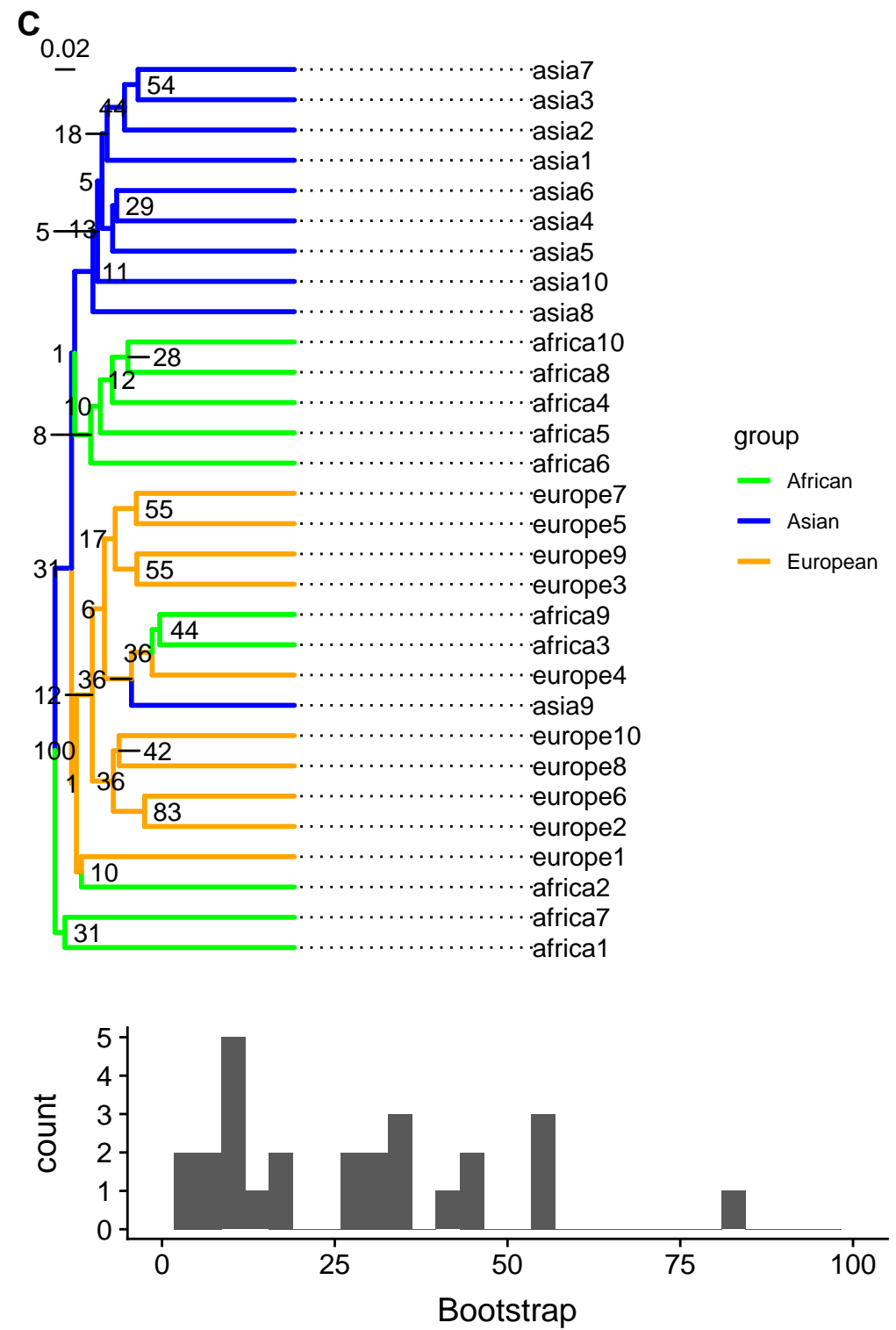

Supplement: S3 Fig — A: time-restricted maps based on human whole-genome sequences; B: regular (non time-restricted) maps based on a simulation of hominin specific demography; C: time-restricted maps based on the same data as B. (PDF) [file pgen.1008449.s009.pdf]
