# Supplementary material for: Inference of recombination maps from a single pair of genomes and its application to ancient samples
Source: PLoS Genet. 2019 Nov 14;15(11):e1008449. doi: 10.1371/journal.pgen.1008449 (PMC6879166; doi:10.1371/journal.pgen.1008449)
Supplement: Attachment — Submitted filename: ResponseToReviewers.odt [file pgen.1008449.s011.odt]

two
Julien Yann Dutheil2019-09-09T15:00:22.367320325JYDAdd the sentences there too
Add the sentences there too
xxx
Reviewer #1: 
1. -On p. 13 the authors claim "these results cannot be accounted for by patterns of LD left by population-specific demographic histories because iSMC infers the recombination landscape while jointly modeling ancestral coalescence rates". I think that this statement conflates two issues, and is misleading. On the one hand, bias introduced by assuming a constant demography (see [1,2,3]; references listed at the end of this review) could cause unrelated populations with similar past population size histories to have similar inferred recombination rates--by accounting for past population sizes iSMC certainly deals with this potential issue. On the other hand, this issue is distinct from the more subtle genealogical LD hinted at in the manuscript. There is some "true" ARG relating all organisms (see [4]), and recombinations that happen in ancient parts of this ARG are likely shared by many extant individuals. The more closely related individuals are, the more likely they are to share these ancient recombination events. This would cause the inferred recombination rates to be correlated, even if the true underlying recombination rates are not (see the discussion in [5]). No method that infers historical recombination rates, including iSMC, can avoid this confounding. And particularly, because iSMC is using the signal from only a pair of haplotypes and so most events are expected to occur on the order of 1 coalescent unit ago (i.e. fairly anciently) many of these events will be shared across different samples, meaning that this confounding should be particularly prevalent in iSMC.

This is a very insightful comment. We added a new batch of simulations to address this issue, and further developed our method to focus on more recent recombination events (see overlapping comment #3 by Reviewer #2).


2. -In a previous version of this manuscript (https://www.biorxiv.org/) the authors included a discussion of the runtime, which is absent in the present manuscript. Any paper presenting a computational method should provide an empirical analysis of the runtime of the method (e.g., how long did it take to perform the whole genome analyses for the humans in the present manuscript?). In general, a runtime of days or weeks is not prohibitive, but users should certainly be aware of what to expect from reading the manuscript. There are also a number of potential speedups to the HMM that while certainly not necessary for publication, could dramatically improve the runtime of iSMC, which would make the method more broadly useful. Specifically, the transition matrix Q_rho has a special structure where conditioned on changing recombination rates, it is equally likely to change to any other recombination rate. In a number of previous methods [6, 7, 8], the exact same structure (although in a different context) has been exploited by computing the transition as a combination of the transition conditioned on changing state (whereby it does not matter which state the HMM was in previously) and conditioned on not changing state (whereby the state can only remain in the same state). Such considerations should reduce the runtime from quadratic to linear in the number of recombination rate bins. Similarly, the genealogical part of the HMM has sufficient structure to reduce the runtime from quadratic to linear (e.g., [9, 10]). An alternative approach is to use the spectral decomposition method of [11], which allows for the "skipping'" of homozygous sites.

We added a paragraph describing computational performance (Methods section). We also thank the reviewer for his careful and helpful suggestions. We are aware that there are computational improvements that would decrease runtime. As noted in the Methods, we are currently using the ziphmm library which creates a data structure based on the sequence of observations, thus helping “skip” regions with homozygous (or missing) sites. Another interesting perspective is to traverse the transition matrix more efficiently, as suggested by the reviewer, as it is for instance done in the aSMC software (Palamara et al. 2018 Nature Genetics). While we have good a priori reasons to believe that such efficient traversal can be extended to the Markov-Modulated case, this requires dedicated investigation.


3. -On pp. 13-14 there is a discussion of why the correlation is higher in simulations than in the real data. While natural selection, introgression, and evolution of the recombination rate certainly play a role, there are simpler reasons for the improved performance on simulated data (discussed below in the minor comments). In particular, the evidence in favor of evolving recombination rates is presented in terms of the patterns of correlation at different scales in the real data and in simulated data. This is not a fair comparison because the r^2 on the simulated data is likely inflated for reasons discussed below and since r^2 is bounded by 1, the slope must necessarily be less steep.

We have now modified this discussion as suggested. 


4. -p. 8 and pp. 10-11, results should be reported for each window size (50kb, 200kb, 500kb, 1Mb), instead of just the median across window sizes on p. 8, and for a single window size for pp. 10-11. Furthermore, since hotspots are on the order of kb in length it would be good to compute these metrics at even finer scales such as 1kb.

We now report medians for different window sizes as suggested. As for 1kb windows, there are several reasons why we do not focus on hotspot mapping (see response to comment #6 below).


5. -p. 10 Saying that the "variance explained is significant" should be removed. Proportion of variance explained is non-negative, and so it only takes on the value 0 if the observed correlation (r) is exactly 0. For continuous observations, this should happen on a set of measure 0, and so no bootstrap-based test would fail to reject this null. A better test would be to see if the bootstrap confidence interval for the correlation itself (i.e., r instead of r^2) contains 0. Furthermore, by providing the observed proportions of variance explained (with confidence intervals) the authors already convey this information.

We thank the reviewer for this suggestion and have modified this section as suggested.


6. -The presented simulation results are biologically unrealistic in such a way that would optimize the performance of iSMC. In particular, in humans and many other species the ratio of the mutation rate to the recombination rate is approximately 1, whereas in the simulations this ratio is >2, which will facilitate inference. This is further exacerbated in the variable mutation rate case where the average mutation rate over the recombination rate is further increased to >10. The length scale of changing recombination rates is also rather unrealistic, with changes occurring either every 100kb or 1Mb on average. Because hotspots tend to be on the order of a few kb in species with PRDM9, the length scales used in simulation are 2-3 orders of magnitude too large to accurately reflect realistic recombination maps. These smoother true recombination maps would make inference substantially easier. Additionally, the simulations involving introgression only test fairly ancient introgression -- for human-like values of Ne and generation time the more recent introgression simulation would correspond to an introgression time of about 70kya. A far more concerning issue in practice would be recent gene flow which would affect longer chunks of the genome, resulting in the sort of long-range LD patterns discussed in the manuscript. It is not absolutely necessary to perform new simulations to account for any of these issues (elevated mutation rate to recombination rate ratio, overly smooth true recombination maps, and ancient introgression events) but it would be good to explicitly point out that these simulation scenarios suggest a much better performance of iSMC than is likely to be seen in practice (see the aforementioned major comment).

The comment about varying mutation rates overlaps with comment #3 from reviewer 3. We added a new batch of simulations to address it by varying the theta / rho ratio. We also agree with the reviewer about the distinction between scales of recombination. We note, however, that our method does not focus on mapping narrow recombination hotspots, hence we do not explore variation at the very fine scale in our simulations. The reasons are as follows. First, we propose a general framework that hopefully is suitable for a wide range of species – including the ones without hotspots. Second, 1-3 kb hotstpots typical of mammals are most likely unidentifiable along a single pair of haploids because the density of SNPs does not allow such resolution. Third, for many population genomic applications, maps at the > 50 kb scale are well suited to explore the effect of recombination. Finally, the high R^2 that we report between iSMC-inferred maps and experimental genetic maps, suggest, in our opinion, that iSMC is relatively robust to the exact a priori distribution of recombination rate.

7. -The performance of the method on data from regions under selection is never benchmarked. While weak background selection can be approximated by a local reduction in Ne in terms of the amount of polymorphism, it is unclear that this should have a monotonic, linear effect on the inferred rho as implied on p. 13. The effect is likely to be even more complex in the presence of recent selective sweeps, stronger background selection, etc... I do not think that this is a major issue or needs to be directly addressed (e.g. by a simulation study), but perhaps a note of caution should be included, especially for species where selection is likely to be pervasive.

We extended the discussion about the potential effect of selection on page 365.


8. -p. 3 "where a survey of the recombination landscape in closely related species are lacking" --> "where a survey of the recombination landscape in closely related species is lacking"

Modified as suggested.


9. -p. 8 "and the short length of blocks with constant [rho] leaves few signal in the data" --> "and the short length of blocks with constant [rho] leaves little signal in the data"

Modified as suggested.


10. -p. 12, the authors discuss dealing with the ancient samples in a different way by first inferring a demography and mean recombination rate, and then using parameters inferred from modern humans as a prior for the posterior decoding. This approach seems reasonable, but it should bias the inferred maps to be closer to modern humans. Since the ancient individuals still form a clade to the exclusion of modern humans, such bias would only imply that Neanderthals and Denisovans have even more diverged recombination maps. It would be good to discuss that this bias is in the "conservative" direction and so it does not affect the major conclusions of this section.

Modified as suggested.


Reviewer #2: 
1. This paper presents a useful new method for jointly inferring population size changes with the recombination rate landscape of a single genome. The method is very well described, and based on benchmarking studies summarized by the authors, it infers maps that are at least as well correlated with pedigree-based maps as those produced by state of the art methods that require input data from many genomes. I do think that although the results presented on pages 10 and 11 are impressive, they don’t seem strong enough to justify the section heading “iSMC outpeforms the state of the art…” Although the point estimate of variance explained by the iSMC is higher than the point estimate of variance explained by LD helmet, the confidence interval of the iSMC estimate includes the LD helmet estimate. Is there a stronger case to be made that the iSMC is doing better than LD helmet overall? Or do certain genomes yield better estimates because those genomes are closely related to the genomes that were used to make the family-based estimates?

We have modified the wording as suggested. We note, however, that all genomes come from the same population (hence relatedness with the strains used to perform the experimental crosses should not be a confounding factor), and that we use the same data for comparison between methods (as pointed out in the manuscript). 


2. One parameter of the method that is not very well described is the gamma prior probability distribution that the recombination rate will change from one locus to the next. There’s nothing in the coalescent model that dictates how fast the recombination rate is likely to change as a function of sequence, so this choice seems somewhat arbitrary and might not be well optimized as is. Unless I missed it, the manuscript doesn’t seem to discuss how the shape and scale of the gamma distribution have been chosen, and it seems worth exploring whether varying them could improve iSMC’s performance. Figure 3E-H suggests that the inferred recombination landscape is systematically too spikey and biased toward high recombination rates, and perhaps this problem could be fixed or reduced by lowering the prior probability of recombination rate change between loci.

We have made the text more explicit and added explanations / definitions in the Methods section. It is now clearly mentioned that the shape of the Gamma distribution (as well as the “stickiness” of the Markov chain) is not fixed, but estimated by the model.


3. It is interesting to see that recombination landscapes cluster within human populations, but I’m concerned that this result could be misleading because of misidentification of genetic drift or demographic change as recombination landscape change. This result would be more convincing if it were accompanied by a proof of concept simulation study. If you were to simulate data from human populations that diverged under a realistic demographic scenario but share the same recombination map, would iSMC infer similar recombination maps from all individuals or instead infer some variation that comes from misidentified demographic variation? In other words, would inferred recombination maps from these populations also appear to cluster by population even though this structure is not built into the simulation?

We thank the reviewer for this suggestion. We added a new batch of simulations to address this concern, and further developed our analysis to show that the signal persists once the confounding effects are dealt with (see results and methods sections). In this new analysis, we exploit iSMC’s explicit modelling of coalescence times to consider only recent recombination events when estimating recombination rates. This has the effect of reducing the impact of shared recombination events due to demography (via the ARG) on the similarity of the maps.


Reviewer #3: 
1. The results from humans are less reliable, with R^2 to the deCODE map of 0.166 at the 50 kb scale and 0.550 at the 1 Mb scale. While the text says that, “This suggests that the differences in R^2 between simulation and case studies are not only driven by noise, but also by biological factors,” this is hard to be certain of. For example, the simulations use higher diversity rates with a genome-wide average theta of 0.015 (pg. 21), or >10-fold higher than human diversity. Since SNPs are the fundamental signal for the analysis, the simulation results need not be informative of the results in humans. Moreover, the deCODE, pedigree-based map is strongly correlated with the LD-based HapMap map (Kong et al. 2010) -- with a correlation (seemingly just R) of 0.729 at the 10 kb scale and 0.920 at 3 Mb.

We would like to point out that the diversity level in the simulations is close to that observed in humans (θ = 0.0012). The referred θ of 0.015 was used exclusively in the set of simulations where we let the mutation rate vary along the genome, for reasons specified therein. Still we understand the reviewer’s concern about levels of diversity (see reply to comment #9) and have addressed it with a new batch of simulations.


2. Given the above, the focus of the manuscript on ancient hominin samples is hard to justify. Instead, the authors could focus on species and potentially ancient samples that have a much higher diversity rate. A related concern is the inferred phylogenies, particularly the locations of the Ust’Ishim ancient sample, which seems more reasonable to place as an out-of-Africa sample, but instead is a sister group to other modern humans.

We understand the reviewer’s concern about the ancient hominins, but we argue that this is a rather “soft focus” that is primarily meant to showcase one of the applications of iSMC that allows investigation in a kind of dataset that was not possible before. Further, we chose hominins because they are well studied and allow us to discuss any possible discrepancies between our results and the existing literature. We added two sentences discussing the unusual position of Ust’Ishim:
“We note that, while Ust’Ishim is branching together with other H. sapiens samples in a well supported group, it is not placed immediately outside the Europe + Asian clade, as expected given that he is part of the out-of-Africa migration. One possibility is that the high level of missing data from this sample increases the noise in the infered recombination map – therefore decreasing the similarity with other H. sapiens maps. Further analyses are necessary to pinpoint the precise reason for this branching pattern.”

3. To help understand the contexts in which iSMC can function, providing more detailed simulation results including a wider range of theta (diversity) values, and potentially expanding on the demographic scenarios tested would be informative.

We added a new batch of simulations to address this concern, varying θ from 0.0006 to 0.0024 (see main text and S2 Fig). iSMC retains high accuracy for realistic values of the ratio theta / rho (the relevant parameter here, as pointed out by the first reviewer) 


5. Lastly, please quote runtimes of iSMC.

We added a dedicated section in the Methods. See also comment #2 by the first reviewer.


6. Pg. 2: “sequences from the ancestral population” - here, “ancestral population” is not well defined and could be clarified.

We have re-written as suggested, with the hope to make this sentence clearer.


7. Pg. 5: “extending the SMC to accommodate spatial heterogeneity in rho ...” - this is about PSMC, not the SMC, right?

Although the implementation of the model focuses on the particular case of the PSMC, the framework we introduce is general and applicable to the SMC as a whole. This is now explicitly mentioned in the first paragraph of the Methods section.


8. Pg. 6: “potentially leading to more accurate estimates” - it seems like the text here could be a bit more specific and conclusive about the estimation accuracy. More specifically, the word “potentially” could be omitted and the context of greater accuracy provided.

Joint-inference should lead to increased accuracy, other things being equal. But it turns out that SMC and LD-based methods are different in many aspects, so if accuracy differs, it could be because of multiple reasons (including sample size). We therefore propose to keep the phrasing that refers to the principle of jointly modelling coalescence rates and the recombination landscape, since the sentence belongs to a section where we introduce the model.


9. Pgs. 8-10: while this is in the Results section, a few more details about the simulation scenarios, such as the meaning of the A (alpha) and g parameters (Fig 2) and what the simulation parameters are would be good to include here. (Also see pg. 12, which mentions rho, alpha, and delta without definition.)

We added a call for the Methods section in the legend of Figure 2. About the description of the simulations (pages 8-10), there is no explicit mentioning of the parameters (we only provide intuition about what the simulation is aiming for), in order to avoid jargon and enhance clarity at this stage of the paper. 
We define the parameters on page 11: “(the genome-wide average recombination rate ρ, the shape of the Gamma distribution α and the transition probability between recombination categories δ)” before referring to them freely on page 12. 


10. Pg. 12 “with 100% bootstrap support (with one exception, see below)” - please give the true % including the exception.

Modified as suggested.


11. Eq. (3): the rho_i notation doesn’t seem to be introduced; only rho_0 and r_j.

The original notation in Eq. 3 stands for rho_1, where 1 indicates the first column of the matrix. Following the reviewer’s comment, we have modified it for clarity (rho_j=1).


12. Eq. (4): the probabilities here, e.g., Pr(rho_l -> rho_m), use a different notation than earlier in the text, where P_lm represents this probability. Would be helpful to be more consistent.

Modified as suggested.


13. Fig. 3: While it seems likely that A-D and E-H represent increasing bin sizes, it would be good to make this more explicit.

Modified as suggested.


14. Pg. 11 / citation 67: This is not the high coverage Vindija Neanderthal paper.

We thank the reviewer for catching this mistake, which we have now corrected.
